# Blood–Brain Barrier Repair of Bevacizumab and Corticosteroid as Prediction of Clinical Improvement and Relapse Risk in Radiation-Induced Brain Necrosis: A Retrospective Observational Study

**DOI:** 10.3389/fonc.2021.720417

**Published:** 2021-10-06

**Authors:** Ruiqi Xue, Meiwei Chen, Jinhua Cai, Zhenhong Deng, Dong Pan, Xiaohuan Liu, Yi Li, Xiaoming Rong, Honghong Li, Yongteng Xu, Qingyu Shen, Yamei Tang

**Affiliations:** ^1^ Department of Neurology, Sun Yat-sen Memorial Hospital, Sun Yat-sen University, Guangzhou, China; ^2^ Department of Radiology, Sun Yat-sen Memorial Hospital, Sun Yat-sen University, Guangzhou, China; ^3^ Guangdong Provincial Key Laboratory of Malignant Tumor Epigenetics and Gene Regulation, Sun Yat-sen Memorial Hospital, Sun Yat-sen University, Guangzhou, China; ^4^ Guangdong Province Key Laboratory of Brain Function and Disease, Zhongshan School of Medicine, Sun Yat-sen University, Guangzhou, China

**Keywords:** blood–brain barrier permeability, bevacizumab, dynamic contrast-enhanced MR imaging, relapse, radiation-induced brain necrosis

## Abstract

**Background:**

Blood–brain barrier (BBB) disruption after endothelial damage is a crucial part of radiation-induced brain necrosis (RN), but little is known of BBB disruption quantification and its role in the evaluation of therapeutic effect and prognosis for drug treatment. In this retrospective study, BBB repair by bevacizumab and corticosteroid and the correlation between BBB permeability and treatment response and relapse were evaluated by dynamic contrast-enhanced MRI (DCE-MRI).

**Methods:**

Forty-one patients with RN after radiotherapy for nasopharyngeal carcinoma (NPC) (28 treated with bevacizumab and 13 with corticosteroid), 12 patients with no RN after NPC radiotherapy, and 12 patients with no radiotherapy history were included as RN, non-RN, and normal groups, respectively. DCE-MRI assessed BBB permeability in white matter of bilateral temporal lobe. DCE parameters were compared at baseline among the three groups. DCE parameters after treatment were compared and correlated with RN volume decrease, neurological improvement, and relapse.

**Results:**

The extent of BBB leakage at baseline increased from the normal group and non-RN group and to RN necrosis lesions, especially *K* ^trans^ (Kruskal–Wallis test, *P* < 0.001). In the RN group, bevacizumab-induced *K* ^trans^ and *v*_e_ decrease in radiation necrosis lesions (both *P* < 0.001), while corticosteroid showed no obvious effect on BBB. The treatment response rate of bevacizumab was significantly higher than that of corticosteroid [30/34 (88.2%) *vs*. 10/22 (45.4%), *P* < 0.001]. Spearman analysis showed baseline *K* ^trans^, *K*_ep_, and *v*_p_ positively correlated with RN volume decrease and improvement of cognition and quality of life in bevacizumab treatment. After a 6-month follow-up for treatment response cases, the relapse rate of bevacizumab and corticosteroid was 10/30 (33.3%) and 2/9 (22.2%), respectively, with no statistical difference. Post-bevacizumab *K* ^trans^ level predicted relapse in 6 months with AUC 0.745 (*P* < 0.05, 95% CI 0.546–0.943, sensitivity = 0.800, specificity = 0.631).

**Conclusions:**

Bevacizumab improved BBB leakage in RN necrosis. DCE parameters may be useful to predict therapeutic effect and relapse after bevacizumab.

## Introduction

Radiation-induced brain necrosis (RN) is a major adverse event in patients after radiotherapy for head and neck tumor. Blood–brain barrier (BBB) disruption is believed to be the major pathological process during the initiation and development of RN (1–3). Ionization results in vascular endothelial damage and BBB permeability increase, which leads to cerebral edema (4, 5). Meanwhile, local necrosis and hypoxia of brain parenchyma induce activation of the hypoxia-induced factor (HIF) pathway and upgrading of vascular endothelial growth factor (VEGF) production (6). VEGF-modulated angiogenesis further aggravates BBB leakage and edema development.

Bevacizumab and corticosteroid have been mainstream drug therapies for RN, with anti-angiogenesis and anti-inflammation effect, respectively, to reduce RN lesions. Corticosteroid with general suppression on inflammation response reduces radiation injury of neurons and endothelial cells and ameliorate demyelination in white matter (7). Bevacizumab restrains angiogenesis and normalizes the microcirculation around RN foci by inhibition of VEGF binding with receptors (8–10). Previous studies have mainly focused on the treatment effect of bevacizumab and corticosteroid on volume decrease of RN necrosis and edema on MRI and clinical improvement of neurological symptoms (11–14). However, the importance of BBB permeability in RN development and recovery was not evaluated in these studies. One previous study has demonstrated that based on the degree of RN volume decrease, bevacizumab shows superiority to corticosteroid with higher treatment response rate and lower relapse rate (12). The fundamental reason for better outcome by bevacizumab is likely associated with BBB leakage repair. Meanwhile, the relationships between BBB permeability and RN volume change, alleviation of neurological injury, and relapse after treatment course all remain undetermined.

Dynamic contrast-enhanced MRI (DCE-MRI), a new non-invasive imaging technique for measurement of tissue microcirculation, has been widely utilized to detect angiogenesis activity by obtaining sequential magnetic resonance images before, during, and after the injection of small molecular gadolinium contrast. DCE-MRI has been affirmed as an imaging marker in tumor microvasculature measurement, especially correlating with tumor detection, prognostic analysis, and VEGF expression (15, 16). Recently, DCE-MRI is also investigated in BBB permeability assessment of various neurological diseases, such as ischemic stroke, cerebral small vessel disease, and Alzheimer’s disease (17–20). Elevation of DCE-MRI parameter level is associated with aggravation of BBB leakage, which is considered to be an important pathophysiological change of the brain (17, 19, 21). These all prompt the potential application value of DCE-MRI in RN, including the evaluation of degree of BBB leakage in RN lesions and evaluation of BBB repair after treatment.

In this study, we aim to 1) quantify and compare the repair of BBB breakdown of bevacizumab *vs*. corticosteroid treatment by DCE-derived parameters, 2) explore the correlation between BBB permeability with clinical improvement (including RN volume decrease, improvement of cognition and quality of life), and 3) explore the prediction efficacy of BBB permeability for RN prognosis after bevacizumab treatment response.

## Methods and Materials

### Study Design

This was a retrospective comparative study. The ethics committee of our hospital approved this study and the requirement for informed consent was waived.

### Patient Selection and Eligibility Criteria

In our institution, 65 patients who underwent DCE-MRI on a 3.0-T clinical MRI system were enrolled as a retrospective DCE-MRI group from September 2017 through December 2018. Forty-one patients, diagnosed with radiation-induced brain injury after radiotherapy for nasopharyngeal carcinoma, were defined as the RN group. In the RN group, 28 patients received bevacizumab (Avastin, Genentech, South San Francisco, CA, USA, 5 mg/kg i.v. every 2 weeks for 4 cycles) and 13 patients received corticosteroid (methylprednisolone 500 mg/day intravenously for three consecutive days and then gradually tapered, followed by 10 mg/day oral prednisone, for 1 month in total). The inclusion criteria were as follows: 1) age >18 years old, 2) radiographic evidence to support the diagnosis of RN in temporal lobe without tumor recurrence or metastases (the diagnosis of RN was defined as hyperintensity edema lesion on T2-weighted imaging and enhanced lesion on post-gadolinium imaging, especially “soap bubble” or “Swiss cheese” enhancement), 3) patients who received standard routine treatment of bevacizumab or corticosteroid for the first time, 4) patients with complete baseline and radiotherapy information, 5) patients who completed clinical score evaluation and DCE-MRI examination before (within 3 days ahead of treatment start) and after the treatment course (within 2 weeks after treatment ending), and 6) routine laboratory studies including urinalysis, complete blood count, liver function, renal function, and coagulation test within a normal range.

The exclusion criteria were as follows: 1) evidence of tumor recurrence or metastasis; 2) treatment routine was not fulfilled due to refusal, loss to follow-up, or severe side effect; 3) evidence of side effect during and after the treatment course including but not limited to active hemorrhage, inadequately controlled hypertension, peripheral neuropathy, and leukopenia for bevacizumab and systemic infection, Cushing’s syndrome, newly diagnosed gastric ulceration, and osteoporosis for corticosteroid; 4) allergy to Gd contrast; and 5) incomplete baseline information, radiotherapy information, and clinical scores.

Twelve patients after nasopharyngeal carcinoma radiotherapy with no RN (defined as the non-RN group) and 12 patients with no radiotherapy history (defined as the normal group), who underwent baseline MR imaging (including DCE-MRI), were included as contrast at baseline after being matched for age and sex proportion with the RN group.

### DCE-MRI Acquisition and Image Analysis

Each RN patient received baseline cranial MR imaging 24–72 h before treatment and follow-up MR imaging 2 weeks after completion of treatment. All cranial MR imaging was performed by the same team including neuroradiologists and medical imaging technologists using the 3.0-T clinical MRI system with a 12-channel head coil (Magnetom Avanto, Siemens Healthcare, Erlangen, Germany). The MR imaging parameters are listed in the **Supplement**.

One neuroradiologist (MC, with 5 years of experience in neuroradiology), blinded to the group assignment, exported DCE MRI images (DICOM files) from the Picture Archiving and Communication System (PACS) and imported them into DCE software [The Medical Imaging Interaction Toolkit, MITK, ITK 4.3.2, VTK 5.10.1, Qt 4.8.7, with Omni Kinetic toolbox (22)]. Extended Tofts linear model was chosen for pharmacokinetic analysis (23–25). The arterial input function (AIF), which described the contrast concentration in blood plasma over time, was sampled from the internal carotid artery by thresholding to determine the earliest contrast uptake (26). Regions of interest (ROIs) of RN and non-RN patients on DCE-MRI were targeted to 1) typical radiation-induced necrosis and edema lesion of the temporal lobe, i.e., necrosis and edema lesion, respectively, corresponded with RN-enhanced lesion on T1-weighted contrast-enhanced imaging and white matter hyperintensity lesion on T2-weighted FLAIR imaging from structural MRI (12) and 2) relatively normal tissue of temporal lobe on the same level of RN lesion. ROIs for imaging of normal patients were targeted to bilateral white matter of temporal lobe. Each ROI included a manual sketch on at least five transection slices, each with an area of 50 mm^2^ on average.

Pharmacokinetic parameters derived from DCE-MRI quantify BBB permeability by contrast redistribution in blood plasma and extravascular extracellular space (EES). According to the extended Tofts linear model, four parameters, namely, *K* ^trans^ (volume transfer constant, min^−1^), *K*_ep_ (flux rate constant between EES and plasma, min^−1^), *v*_e_ (volume fraction of EES), and *v*_p_ (volume fraction of blood plasma), were calculated pixel-by-pixel in each ROI and then averaged among five slices. The complete process of DCE-MRI image is shown in **Supplement Figure 1**.

To calculate RN volume on structural MRI, coronary images of T1-weighted contrast-enhanced images and T2-weighted FLAIR images were extracted as DICOM files. One neurologist (RX), blinded to the group assignment, processed the images with ITK-SNAP software (version 3.8.0) for structure delineation and semiautomatic segmentation. The volume of RN necrosis and edema lesion was measured by pixels.

### Scale Assessment

Patients in the RN and non-RN groups completed cognitive assessment by Montreal Cognitive Assessment (MoCA, Chinese version) (27), symptomatic assessment of radiation injury by the Late Effects of Normal Tissue (LENT)/Subjective, Objective, Management, Analytic (SOMA) scales (28), and assessment of quality of life by the brief version of World Health Organization Quality of Life Instrument/Short Version (WHOQOL-BREF) scale (29) before and after the treatment course as standard of care. The corresponding scales were performed by experienced doctors specialized in neuropsychological evaluation at the baseline and the end of the treatment course.

### Statistical Analysis

The Shapiro–Wilk test was used to assess the normality of continuous variables. Clinical characteristics of the normal, non-RN, and RN groups were compared with *χ* ^2^ test for categorical variables and Student’s *t*-test or Wilcoxon test for continuous variables. DCE-MRI parameters were calculated with log_10_ (parameter median) and were compared 1) among white matter of the normal group, white matter of the non-RN group, RN edema, and RN necrosis at baseline with Mann–Whitney *U* test and 2) among RN edema and RN necrosis before and after bevacizumab or corticosteroid treatment with paired Wilcoxon test. Decreased percentage of RN volume on T2 FLAIR ≥25% was defined as treatment response and <25% as non-response (12, 14). The increased percentage of RN volume on T2 FLAIR ≥10% compared with the last MRI after treatment reaction was defined as relapse and <10% as non-relapse (12). The treatment response rate equaled to the number of RN foci with treatment response/total number of RN foci. The relapse rate equaled to the number of RN foci with relapse/number of RN foci with treatment response. Subgroup analysis was made among treatment reaction/non-reaction subgroup from the RN group and relapse/non-relapse subgroup after bevacizumab. Receiver operating characteristic (ROC) analysis was performed to assess the prediction efficacy of relapse by DCE parameters, with the area under the ROC curve (AUC) and Youden index for optimal cutoff value. Correlation analysis between baseline DCE parameters, RN volume change, and scale score improvement was conducted. Statistical analysis and visualization were performed with R software (version 3.6.0), and ROC analysis was performed with pROC package (30). *P*-value <0.05 was considered to indicate statistical significance.

## Results

### Baseline Characteristics of Patients in the RN, Non-RN, and Normal Groups

**Table 1** summarizes the baseline information of patients in the RN, non-RN, and normal groups. Variables among the three groups showed no significant difference except for gender proportion. **Supplement Table 1** summarizes the baseline characteristics of patients treated with bevacizumab and corticosteroid within the RN group. Most of the variables showed balance at baseline, while distribution of primary tumor stage, radiotherapy dose of neck, and the interval between diagnosis of RN and the first in-hospital treatment in our institution (IBT) between two treatments showed statistical difference.

**Table 1:** Baseline comparison of the RN, non-RN, and normal groups.

	RN	Non-RN	Normal	*P*-value
**Total**	41	12	12	
**Gender**				
Male	34 (82.9)	9 (75.0)	5 (41.7)	0.017
Female	7 (17.1)	3 (25.0)	7 (58.3)	
**Age**	47.9 (9.5)	55.3 (11.3)	52.0 (14.6)	0.096
**Smoking history**				
Without	34 (82.9)	11 (91.7)	12 (100.0)	0.256
With	7 (17.1)	1 (8.3)	0 (0.0)	
**MoCA**	24.0 (21.0, 26.0)	20.5 (16.0, 25.2)	–	0.124
**LENT/SOMA**	8.0 (6.0, 12.0)	11.5 (6.8, 22.8)	–	0.277
WHOQOL	88.8 (13.8)	90.9 (11.1)	–	0.626
**T**			–	
1	1 (2.4)	0 (0.0)		0.905
2	3 (7.3)	1 (8.3)		
3	17 (41.5)	6 (50.0)		
4	20 (48.8)	5 (41.7)		
**N**			–	
0	7 (17.1)	1 (8.3)		0.481
1	18 (43.9)	8 (66.7)		
2	13 (31.7)	3 (25.0)		
3	3 (7.3)	0 (0.0)		
**Stage**			–	
1	1 (2.4)	0 (0.0)		0.517
2	15 (36.6)	2 (16.7)		
3	16 (39.0)	7 (58.3)		
4	9 (22.0)	3 (25.0)		
**Radiotherapy methods**			–	
Conventional	11 (26.8)	3 (25.0)		1.000
IMRT	30 (73.2)	9 (75.0)		
***D*_max_ of the temporal lobe (Gy)**	70.0 (68.0, 70.0)	70.0 (68.0, 70.0)	–	0.769
**Total dose of the neck (Gy)**	60.0 (54.0, 66.0)	57.0 (50.0, 64.5)	–	0.643
**Chemotherapy**			–	
Without	13 (31.7)	2 (16.7)		0.514
With	28 (68.3)	10 (83.3)		
**Secondary radiotherapy**			–	
Without	34 (82.9)	9 (75.0)		0.843
With	7 (17.1)	3 (25.0)		
**Radiation injury of CN**			–	
Without	28 (68.3)	4 (33.3)		0.065
With	13 (31.7)	8 (66.7)		

Data are presented as mean (SD), median (IQR), or N (%).

MoCA, Montreal Cognitive Assessment; LENT/SOMA, the Late Effects of Normal Tissue (LENT)/Subjective, Objective, Management, Analytic (SOMA); WHOQOL, the World Health Organization Quality of Life Instrument/Short Version (WHOQOL-BREF); Dmax of the temporal lobe, the maximum radiation dose of the temporal lobe; IMRT, intensity-modulated radiation therapy; IRB, the interval between radiotherapy and brain necrosis; Radiation injury of CN, cranial nerve injury due to radiation.

### Comparison of DCE Parameters Among the RN, Non-RN, and Normal Groups

**Figure 1:**
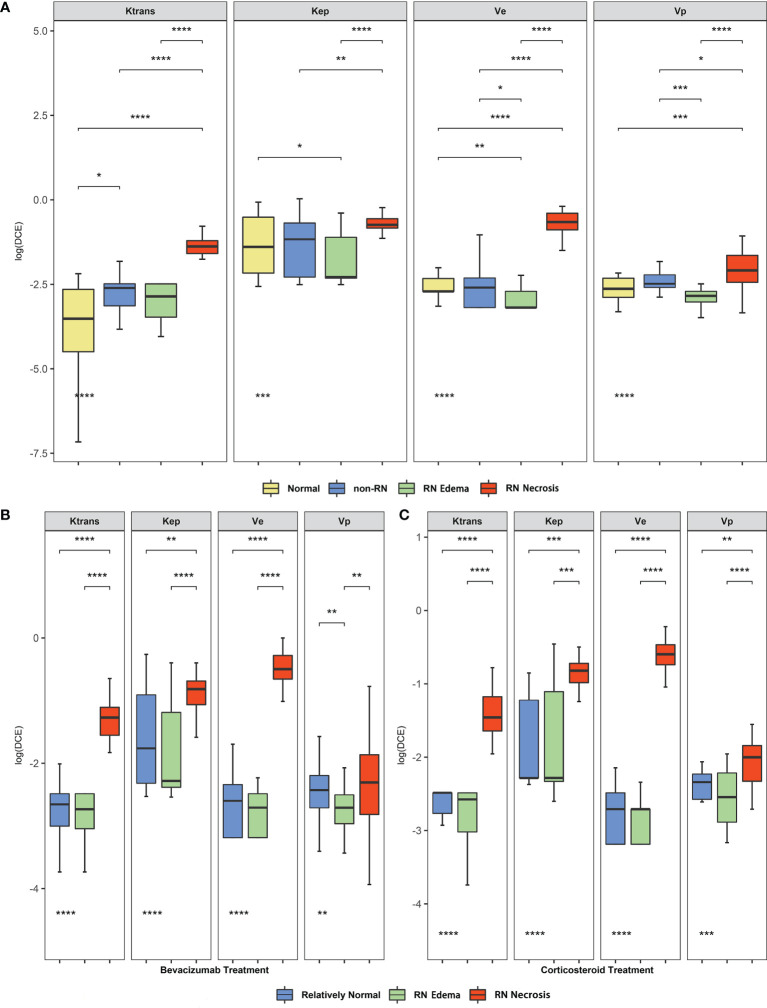
**(A)** Comparison of dynamic contrast-enhanced (DCE)-derived parameters between the normal, non-RN, and normal groups. **(B, C)** Comparison of DCE-derived parameters among radiation-induced brain necrosis (RN) necrosis, edema lesions, and relatively normal white matter before bevacizumab and corticosteroid in the radiation injury group. The *P*-values marked below indicate the results of the Mann–Whitney *U* test. *P value < 0.05, **P value < 0.01, ***P value < 0.001, ****P value < 0.0001.

An obvious difference of DCE parameters among the normal group, non-RN group, and RN group was found in this study. In **Figure 1A**, four DCE-derived parameters all showed significant difference between white matter of the normal group, white matter of the non-RN group, and RN necrosis lesions, especially in *K* ^trans^ (Kruskal–Wallis test, *P* < 0.001, non-RN *vs*. normal, *P* < 0.05 and RN necrosis *vs*. non-RN, *P* < 0.001, **Figure 1A**), representing increasing BBB leakage from the normal group to the non-RN group and to RN necrosis. Compared with the non-RN and normal groups, edema lesions showed relatively low levels of parameters in *K*_ep_, *v*_e_, and *v*_p_, especially in *v*_e_ (RN edema *vs*. non-RN, *P *< 0.05 and RN edema vs. normal, *P* < 0.01, respectively, **Figure 1A**).

### Comparison of DCE Parameters Before and After Bevacizumab or Corticosteroid for BBB Repair Effect

We compared the baseline level of DCE-derived parameters of bevacizumab and corticosteroid treatment in the RN group. In **Figures 1B, C**, *K* ^trans^, *K*_ep_, *v*_e_, and *v*_p_ in RN necrosis lesion were significantly higher than those of RN edema lesions and relatively normal region (Kruskal–Wallis test, all *P* < 0.01, *K* ^trans^ both *P* < 0.001, *K*_ep_ *P* < 0.001 and *P* < 0.01, *v*_e_ both *P* < 0.001, *v*_p_ both *P* < 0.01, **Figures 1B, C** **)**, indicating that BBB damage of RN necrosis was the most prominent in the whole radiation lesion. Parameters of RN edema lesions were lower than the relatively normal region with no statistical difference except for *v*_p_ in the bevacizumab treatment group (*v*_p_ *P* < 0.01, **Figure 1B**).

**Figure 2:**
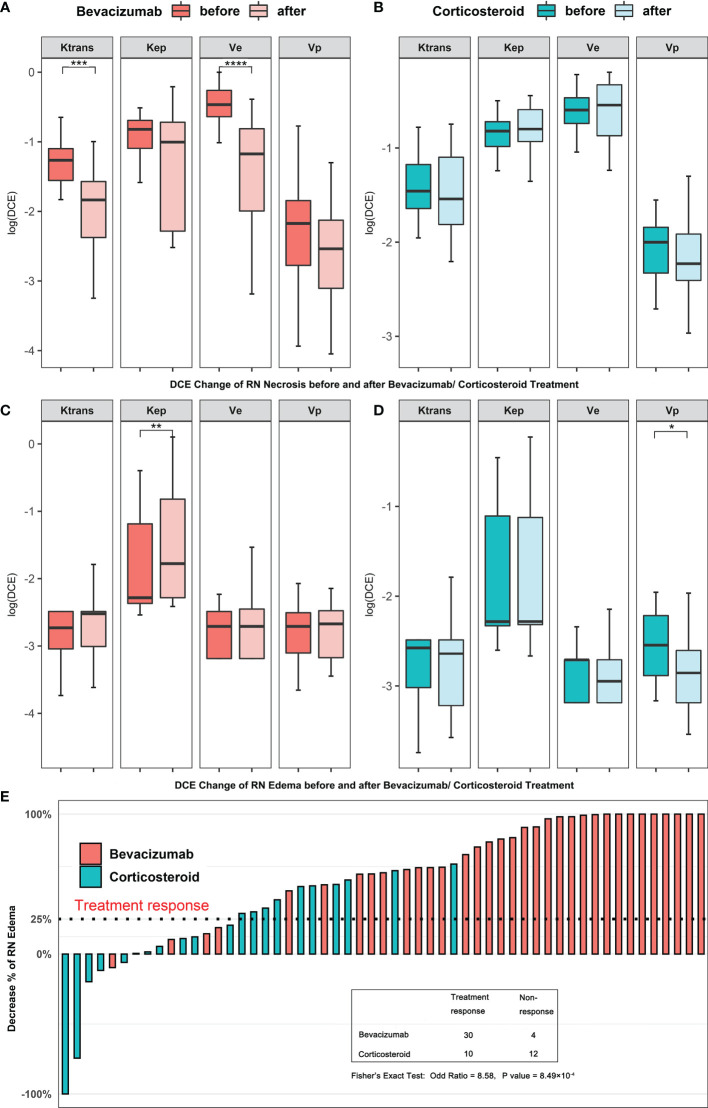
**(A–D)** Comparison of DCE-derived parameters of RN before and after bevacizumab **(A, C)** or corticosteroid **(B, D)** treatment. **(E)** Treatment response cases in two treatment groups. The straight dash line indicates treatment response. *P value < 0.05, **P value < 0.01, ***P value < 0.001, ****P value < 0.0001.

**Figure 3:**
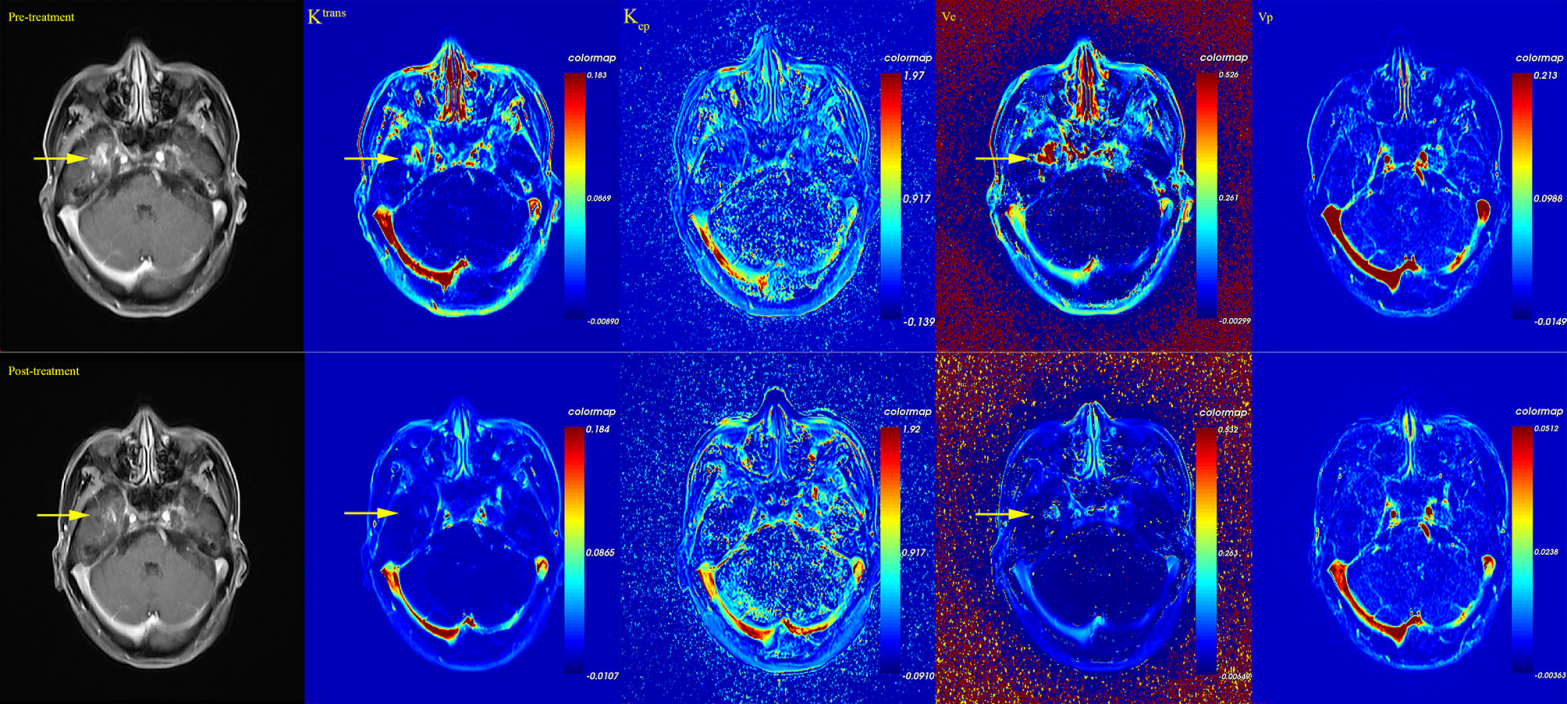
Pseudo-color map of DCE-derived parameters of one patient before and after bevacizumab treatment. After bevacizumab, a decrease of RN enhancement and a decrease of *K* ^trans^ and *v*_e_ level were observed.

Then, we studied the alteration of DCE parameter level before and after bevacizumab or corticosteroid treatment and compared the treatment reaction rate. In **Figures 2A, B**, for RN necrosis lesions, *K* ^trans^ and *v*_e_ levels decreased significantly after bevacizumab treatment (Wilcoxon test, *K* ^trans^ −1.265 *vs*. −1.835, *P* < 0.001 and *v*_e_ −0.466 *vs*. −1.173, *P* < 0.001, **Figures 2A, B** **)**, and no significant change was observed for corticosteroid treatment. In **Figures 2C, D**, for RN edema lesions, *K*_ep_ increased after bevacizumab and *v*_p_ decreased after corticosteroid (Wilcoxon test, *K*_ep_ −2.283 *vs*. −1.777, *P* < 0.01 and *v*_p_ −2.545 *vs*. −2.855, *P* < 0.05, **Figures 2C, D** **)**. Pseudo-color map of typical DCE-derived parameter change before and after bevacizumab is listed in **Figure 3**. After bevacizumab treatment, signal intensity of *K* ^trans^ and *v*_e_ distinctly attenuated along with decrease of RN volume on the pseudo-color map, which indicated that bevacizumab could alleviate BBB leakage and reduce angiogenesis.

### Correlation Analysis Between DCE Parameters and Clinical Improvement

We found a significant difference in the treatment response rate in the two treatments [bevacizumab 30/34 (88.2%) *vs*. corticosteroid 10/22 (45.4%), Fisher’s exact test, *P* < 0.001, **Figure 2E**]. Considering the alteration of DCE parameters and treatment response rate together, we assumed that bevacizumab showed therapeutic superiority to corticosteroid in that bevacizumab prominently repaired BBB leakage in RN lesions.

We further compared *K* ^trans^ and *v*_e_ levels of RN necrosis in the treatment response and non-response subgroups of bevacizumab and corticosteroid. In treatment response cases, *K* ^trans^ and *v*_e_ levels of post-bevacizumab subgroup decreased significantly compared with pre-bevacizumab (paired Wilcoxon test, *K* ^trans^ −1.835 *vs*. −1.271, *P* < 0.001 and *v*_e_ −1.187 *vs*. −0.506, *P* < 0.001, **Supplement Figures 2A, B** **)** and post-corticosteroid subgroups (unpaired Wilcoxon test, *K* ^trans^ −1.835 *vs*. −1.534, *P* = 0.019 and *v*_e_ −1.187 *vs*. −0.575, *P* < 0.01, **Supplement Figures 2A, B** **)**. In non-response cases, *K* ^trans^ and *v*_e_ levels of post-bevacizumab subgroup decreased compared with pre-bevacizumab, but with no statistical difference; no significant difference was found among the pre-/post-corticosteroid subgroups.

**Figure 4:**
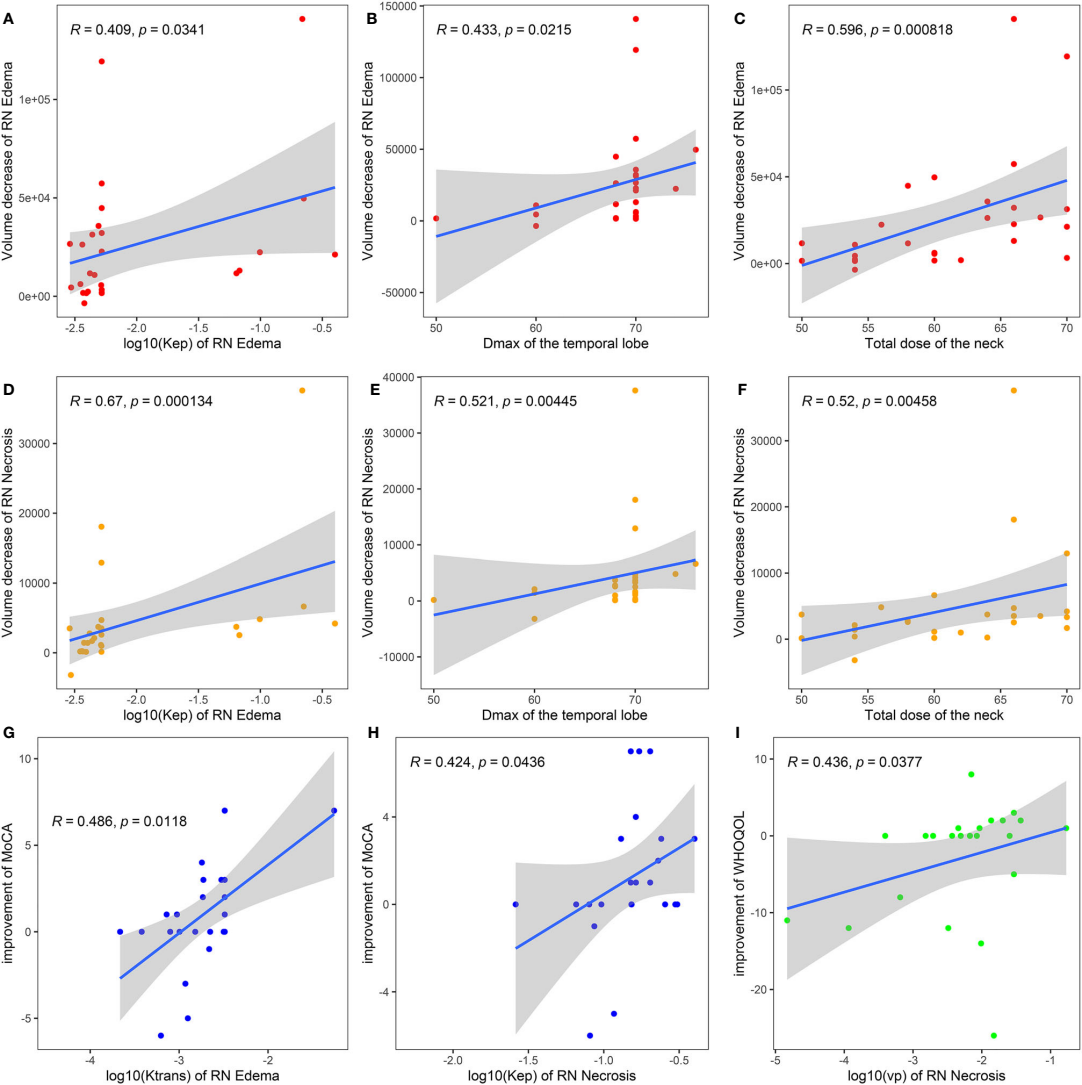
Spearman correlation analysis of baseline DCE parameters of bevacizumab treatment with volume decrease of RN edema **(A–C)**, RN necrosis **(D–F)**, and symptomatic improvement scaled by clinical scores **(G–I)**. *D*_max_ of the temporal lobe, the maximum radiation dose of the temporal lobe; MoCA, Montreal Cognitive Assessment; LENT/SOMA, the Late Effects of Normal Tissue (LENT)/Subjective, Objective, Management, Analytic (SOMA); WHOQOL, the World Health Organization Quality of Life Instrument/Short Version (WHOQOL-BREF).

We also studied the relationship between baseline DCE parameter and the clinical improvement measured by RN volume and scale scores. Spearman correlation coefficient results are shown in **Figure 4**. *K*_ep_ of RN edema, *D*_max_ of the temporal lobe, and total dose of the neck positively correlated with volume decrease of RN edema lesions (Spearman coefficient 0.409, 0.433, 0.596, *P* < 0.05, **Figures 4A** **–C**) and RN necrosis lesions (Spearman coefficient 0.670, 0.521, 0.520, *P* < 0.01, **Figures 4D** **–F**). *K* ^trans^ of RN edema and *K*_ep_ of RN necrosis positively correlated with improvement of MoCA (Spearman coefficient 0.486 and 0.424, *P* < 0.05, **Figures 4G, H** **)**. *V*_p_ of RN necrosis positively correlated with improvement of WHOQOL (Spearman coefficient 0.436, *P* < 0.05, **Figure 4I**).

### Predictive Efficacy of DCE Parameters for RN Relapse After Bevacizumab Treatment Response

**Figure 5:**
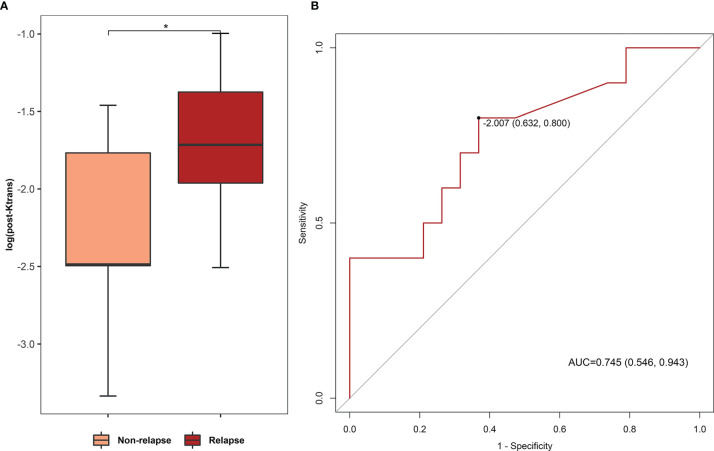
**(A)** Comparison of post-bevacizumab *K* ^trans^ levels between non-relapse and relapse groups. **(B)** ROC analysis of post-bevacizumab *K* ^trans^ level for relapse prediction. (AUC 0.745, *P* < 0.05, 95% CI 0.546–0.943, sensitivity = 0.800, specificity = 0.631; cutoff value based on Youden index: log_10_ *K* ^trans^ = −2.01). *P value < 0.05.

We followed up the treatment response cases in bevacizumab and corticosteroid treatment for 6 months for comparison of relapse rate, and we found that post-bevacizumab *K* ^trans^ level of RN necrosis predicted RN relapse in a 6-month follow-up. Among treatment response cases, 10 of 30 (33.3%) RN foci showed relapse after bevacizumab, while 2 of 9 RN foci (22.2%, with one case lost to follow-up) showed relapse after corticosteroid. Relapse rates between two treatments showed no statistical difference (Fisher’s exact test, *P* > 0.05). We compared post-bevacizumab DCE parameter levels of RN necrosis between the relapse group and the non-relapse group and noticed that *K* ^trans^ acts as a prediction for relapse. In **Figure 5**, post-bevacizumab *K* ^trans^ level showed a significant difference between the non-relapse and relapse groups (Wilcoxon test, *K* ^trans^ −2.487 *vs*. −1.715, *P* = 0.034, **Figure 5A**). ROC analysis of post-bevacizumab *K* ^trans^ level showed that AUC was 0.745 (*P* < 0.05, 95% CI 0.546–0.943, sensitivity = 0.800, specificity = 0.631), indicating a favorable predictive efficacy. The cutoff value of log_10_ *K* ^trans^ was −2.01, i.e., the cutoff value of *K* ^trans^ was 9.77 × 10^−3^ (**Figure 5B**). Thus, *K* ^trans^ of RN necrosis <9.77 × 10^−3^ in patients with effective response after bevacizumab treatment would indicate less likely relapse in 6 months.

## Discussion

Here, we compared the repair effect of bevacizumab and corticosteroid on BBB damage in RN by DCE-MRI evaluation and explored the correlation between BBB permeability and clinical improvement and prognosis after bevacizumab treatment. We found that RN necrosis lesions had the most severe BBB leakage (typically, the *K* ^trans^ parameter) compared with RN edema lesions and relatively normal region. Bevacizumab induced decrease of *K* ^trans^ and *v*_e_ level, thus relieving BBB leakage in RN necrosis lesions. Meanwhile, bevacizumab decreased the RN volume with high treatment response rate. On the contrary, corticosteroid showed no obvious repairment on BBB and reduced RN volume to a limited extent. When two treatment strategies both reached the reactive level, bevacizumab showed superiority to corticosteroid with BBB leakage repair on RN necrosis. Baseline *K* ^trans^ and *K*_ep_ positively correlated with RN volume decrease and cognition improvement in patients treated with bevacizumab. Thus, a high level of baseline *K* ^trans^ and *K*_ep_ in bevacizumab treatment might indicate relatively better clinical improvement in RN volume decrease, cognition, and quality of life. In addition, post-bevacizumab *K* ^trans^ level of RN necrosis would act as an imaging predictor for RN relapse in 6 months.

Our study elucidated that RN necrosis lesions, i.e., enhanced lesions on T1-weighted contrast imaging, undertook the most obvious BBB leakage with a high level of *K* ^trans^ and *v*_e_, which was the target of bevacizumab for BBB repair. Based on pathological study, RN necrosis lesions contain local micronecrosis foci (necrosis of neuron, glia, endothelial cells, etc.) with activated astrocytes and microglia around (2, 3, 10). Activated astrocytes strongly express VEGF-A and induce angiogenesis, which causes progressive increase of BBB permeability. Thus, RN necrosis with severe BBB leakage manifests as enhancement on structural T1-weighted contrast-enhanced and DCE-MRI imaging. In our study, bevacizumab targeted to block the VEGF-A secreted by activated astrocytes around micronecrosis foci and manifested as repair of BBB leakage, which turned out to lower down DCE parameters in the whole RN necrosis lesion (31, 32). Corticosteroid mainly acted as an anti-inflammation agent with no direct effect on VEGF-A and angiogenesis, so it was reasonable that no obvious alteration of DCE parameters was observed after corticosteroid treatment. RN edema lesions, on the contrary, were vasogenic edema after BBB leakage in RN necrosis and neuroinflammation (10). No obvious BBB leakage occurred in edema lesions per se, so no difference of DCE parameters between RN edema and relatively normal white matter could be interpretable. RN edema lesions could not be directly eliminated by bevacizumab or corticosteroid, and BBB permeability of edema lesions was not altered. So, no transparent alteration of BBB parameters was observed in RN edema lesions after bevacizumab and corticosteroid treatment.

In our study, *K* ^trans^ and *v*_e_ quantified the repair effect of bevacizumab. *K* ^trans^ is defined as the number of contrast particles that are distributed to the interstitium per unit of time, tissue volume, and arterial plasma concentration (25, 33). In RN, when BBB progressively losses integrity and angiogenesis aggravates, blood components extravasate through dysfunctional endothelial cells into EES, thus carrying more Gd contrast into EES and increasing *K* ^trans^. *v*_e_ indicates the interstitial space for contrast extravasation, i.e., the leakage space. Both *K* ^trans^ and *v*_e_ are generally interpreted as microcirculation permeability in previous research studies (34, 35). In our study, descending of *K* ^trans^ and *v*_e_ in bevacizumab treatment, especially in bevacizumab response cases, was interpreted as improvement of BBB leakage and reduction of RN-induced angiogenesis by bevacizumab treatment.

High relapse rate has been observed in a previous study of RN drug treatment. About 29.1% patients with bevacizumab treatment response showed RN recurrence on MRI in a 6-monthsfollow-up in previous studies; however, no biomarker has been found as an indicator of relapse risk (12).This problem restrained the effectiveness and utility of bevacizumab in clinical practice. Our study for the first time found a useful indicator in terms of BBB permeability; that is, post-bevacizumab *K* ^trans^ level of RN necrosis could differentiate the relapse group from the non-relapse group. ROC analysis showed good performance of post-treatment *K* ^trans^ for RN relapse prediction and the sensitivity and specificity reached 0.800 and 0.631, respectively. If post-bevacizumab *K* ^trans^ in RN necrosis is reduced below the cutoff value, RN relapse will be less likely to occur in 6 months. This further emphasized the importance of BBB permeability in both the evaluation of therapeutic effect and the prediction of relapse in RN treatment.

There are several limitations in this study. First, limitations in DCE-MRI technology may affect the results in our study. On one hand, the DCE-MRI pharmacokinetic model is utilized on the basis of distribution of Gd contrast concentration in circulation. Thus, hemodynamic factors, such as cardiac output, blood pressure, and velocity of blood flow, will influence the calculation of DCE-MRI parameters. This can be corrected by consistent choice of blood vessel for AIF and measurement of hemodynamic features by transcranial Doppler ultrasound. On the other hand, no gold standard or widely accepted manipulation for DCE-MRI has been proposed, so radiological parameters can exert impact on the temporal and spatial resolution in DCE-MRI (36). Second, selection biases may exist in our study due to the retrospective nature, thus incurring imbalance factors in different groups. Although bevacizumab has been proved to be superior than corticosteroids by decreasing the necrosis volume significantly (12), corticosteroid, as a traditional and commonly accepted therapy, is still placed as the first choice in RN patients with high hemorrhagic tendency, history of thrombosis, RN relapse, and intolerance of other treatments. Such situation will, to some extent, influence the patient selection in our study. Third, as a clinical observational study, we did not further confirm the therapeutic effect of bevacizumab and the causality between BBB repair and symptomatic improvement on biological specimens or animal models. Fourth, the relatively small sample size may affect the generality of conclusions in this study. A study on BBB permeability of RN with a large population cohort will be necessary in the future.

## Conclusions

Bevacizumab alleviated BBB leakage and decreased the RN volume with high treatment response rate compared with corticosteroid. DCE-derived parameters, especially *K* ^trans^, can be used as useful imaging indicators to evaluate BBB permeability, reflect clinical improvement, and predict lesion relapse in bevacizumab treatment. Therefore, the therapeutic significance of bevacizumab for RN should be more emphasized. Adding DCE sequence into standard cranial MRI examination of RN will facilitate assessment of BBB leakage and treatment response of bevacizumab. Moreover, post-bevacizumab DCE-MRI results hint a duration of effectiveness of bevacizumab and predict a 6-month relapse risk, thus boosting precise treatment in RN.

Recently, DCE-MRI has been proved useful in the study of glymphatic efflux and cognition in neurodegenerative diseases (37, 38). Therefore, a prospective study with a large population on BBB permeability of RN as well as glymphatic function will be necessary in the future, in order to unravel the alteration of waste elimination through glymphatics and cognition after radiation injury of the brain.

## Data Availability Statement

The original contributions presented in the study are included in the article/**Supplementary Material**. Further inquiries can be directed to the corresponding authors.

## Ethics Statement

This was a retrospective comparative study. The ethics committee of our hospital approved this study and the requirement for informed consent was waived.

## Author Contributions

Conception and design: QS and YT. Acquisition of data: RX, MC, ZD, DP, and XL. Analysis and interpretation of data: RX, JC, MC, and HL. Writing, review, and/or revision of the manuscript: all authors. Study supervision: QS and YT. All authors contributed to the article and approved the submitted version.

## Funding

This work was supported by the National Natural Science Foundation of China (81925031, 81820108026), and the Science and Technology Program of Guangzhou (202007030001) to YT; the Science and Technology Planning Project of Guangzhou (201704030033) and the National Natural Science Foundation of China (81872549) to YL; the National Natural Science Foundation of China (82003389) to HL; and the Youth Program of National Natural Science Foundation of China (81801229) to YX.

## Conflict of Interest

The authors declare that the research was conducted in the absence of any commercial or financial relationships that could be construed as a potential conflict of interest.

## Publisher’s Note

All claims expressed in this article are solely those of the authors and do not necessarily represent those of their affiliated organizations, or those of the publisher, the editors and the reviewers. Any product that may be evaluated in this article, or claim that may be made by its manufacturer, is not guaranteed or endorsed by the publisher.
