# Supplementary material for: Blood–Brain Barrier Repair of Bevacizumab and Corticosteroid as Prediction of Clinical Improvement and Relapse Risk in Radiation-Induced Brain Necrosis: A Retrospective Observational Study
Source: Front Oncol. 2021 Oct 6;11:720417. doi: 10.3389/fonc.2021.720417 (PMC8526720; doi:10.3389/fonc.2021.720417)
Supplement: Supplementary file 1 [file DataSheet_1.docx]

Supplementary Material:

Blood-brain Barrier Repairment of Bevacizumab and Corticosteroid As Prediction of Clinical Improvement and Relapse Risk in Radiation-induced Brain Necrosis: A Retrospective Observational Study

DCE-MRI imaging parameters

The MR imaging examination included standard T1-weighted (20 slices; TSE; repetition time/ echo time [TR/TE], 1420/8.4 ms), T2-weighted (20 slices; TSE; TR/TE, 2300/90 ms), fluid-attenuated inversion recovery (FLAIR) (20 slices; TSE; TR/TE, 8000/90 ms), and contrast-enhanced T1-weighted imaging (20 slices; FLASH_FS; TR/TE, 138/5 ms), and DCE imaging.

T1-weighted DCE perfusion imaging with 3D T1-weighted volume interpolated body examination (T1W-VIBE) sequences was acquired by the following parameters: 48 slices, TR/TE, 5.3/2.5 ms, flip angle, 10°, 3 mm slice thickness, no gap, matrix size, 288×198, and total acquisition time, 6 minute 24 seconds. 48 layers were acquired to cover entire volume of brain. Before DCE acquisition, multiple flip angle images (2°, 4°, 6°, 8°, 10° and 12°) were obtained for the calculation of T1 maps using the same sequence and parameters except for the flip angle.

Intravenous administration of Gadolinium contrast (Omniscan, GE Healthcare, Ireland) was performed 0.1 mmol/kg of body weight at a rate of 3.5 mL/sec by a power injector (Spectris; Medrad, Pittsburgh, Pa), and a 20 mL bolus injection of saline followed at the same injection rate.

**SUPPLEMENT TABLE 1** Baseline of patients in bevacizumab and corticosteroid treatment of RN group

|  | **RN** | | **P-value** |
| --- | --- | --- | --- |
|  | **Bevacizumab** | **Corticosteroid** |  |
| **Total** | 28 | 13 | — |
| **RN foci** | 34 | 22 | — |
| **Gender** |  |  |  |
| Male | 25 (89.3) | 9 (69.2) | 0.253 |
| Female | 3 (10.7) | 4 (30.8) |  |
| **Age** | 46.5 (10.3) | 50.8 (6.9) | 0.183 |
| **Smoking history** |  |  |  |
| Without | 23 (82.1) | 1. 84.6) | >0.999 |
| With | 5 (17.9) | 2 (15.4) |  |
| **MoCA** | 24.0 | 22.0 | 0.108 |
|  | (21.8, 27.0) | (19.0, 24.0) |  |
| **LENT.SOMA** | 10.0 | 8.0 | 0.877 |
|  | (5.8, 12.8) | (6.0, 12.0) |  |
| **WHOQOL** | 91.4 (10.8) | 83.2 (18.0) | 0.079 |
| **T** |  |  |  |
| 1 | 1 (3.6) | 0 (0.0) | 0.302 |
| 2 | 2 (7.1) | 1 (7.7) |  |
| 3 | 14 (50.0) | 3 (23.1) |  |
| 4 | 11 (39.3) | 9 (69.2) |  |
| **N** |  |  |  |
| 0 | 5 (17.8) | 2 (15.4) | 0.632 |
| 1 | 12 (42.9) | 6 (46.1) |  |
| 2 | 8 (28.6) | 5 (38.5) |  |
| 3 | 3 (10.7) | 0 (0.0) |  |
| **Stage** |  |  |  |
| 1 | 1 (3.6) | 0 (0.0) | <0.001 |
| 2 | 14 (50.0) | 1 (7.7) |  |
| 3 | 13 (46.4) | 3 (23.1) |  |
| 4 | 0 (0.0) | 9 (69.2) |  |
| **Radiotherapy methods** | |  |  |
| Conventional | 7 (25.0) | 4 (30.8) | 0.993 |
| IMRT | 21 (75.0) | 9 (69.2) |  |
| **Dmax of the temporal lobe (Gy)** | 70.0  (68.0, 70.0) | 70.0  (68.0, 70.0) | 0.950 |
| **Total dose of the neck (Gy)** | 60.0  (54.0, 66.0) | 54.0  (50.0, 60.0) | 0.042 |
| **Chemotherapy** |  |  |  |
| Without | 12 (42.9) | 1 (7.7) | 0.059 |
| With | 16 (57.1) | 12 (92.3) |  |
| **Secondary radiotherapy** |  |  |  |
| Without | 23 (82.1) | 11 (84.6) | >0.999 |
| With | 5 (17.9) | 2 (15.4) |  |
| **Radiation Injury of CN** |  |  |  |
| Without | 19 (67.9) | 9 (69.2) | >0.999 |
| With | 9 (32.1) | 4 (30.8) |  |
| **IRB (month)** | 42.5  (27.3, 56.7) | 37.9  (32.3, 44.3) | 0.575^†^ |
| **IBT (month)** | 3.8  (1.4, 9.3) | 15.6  (6.1, 34.5) | 0.013^‡^ |
| **Treatment response**  Without | 4 (11.8) | 12 (54.5) | 0.727 |
| With | 30 (88.2) | 10 (45.5) |  |

Moca, Montreal Cognitive Assessment; LENT/SOMA, the Late Effects of Normal Tissue (LENT)/Subjective, Objective, Management, Analytic (SOMA); WHOQOL, the World Health Organization Quality Of Life Instrument/Short Version (WHOQOL-Bref); Dmax of the temporal lobe, the maximum radiation dose of the temporal lobe; IMRT, intensity-modulated radiation therapy; Radiation injury of CN, cranial nerve injury due to radiation; IRB, the interval between radiotherapy ending and the diagnosis of RN; IBT, the interval between the diagnosis of RN and the first bevacizumab/corticosteroid treatment in our institution.

Data are presented as mean (SD), median (IQR), or N (%).


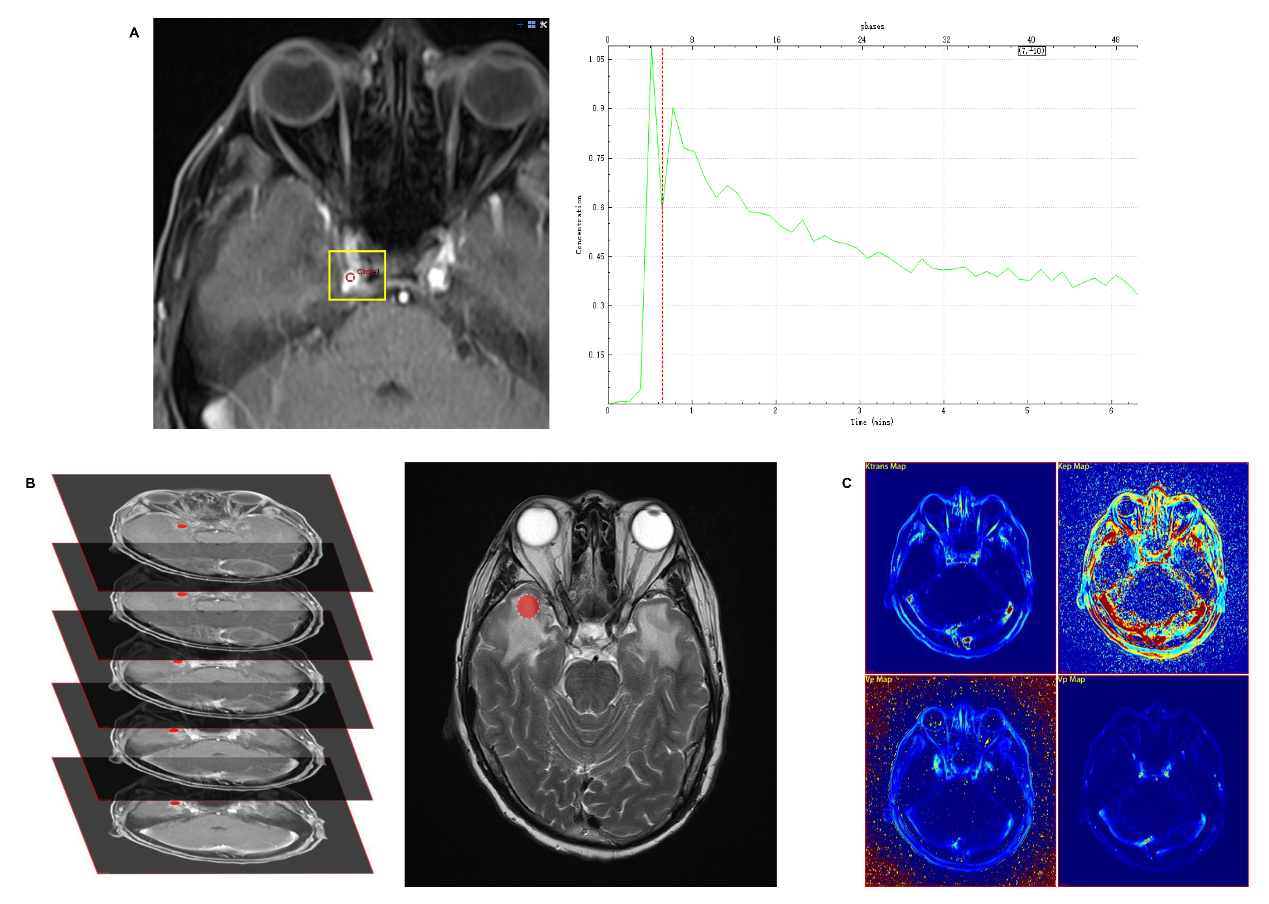


**SUPPLEMENT FIGURE 1** Illustration of DCE-derived parameter measurement. A typical process of DCE-derived parameter measurement by pharmacokinetic model includes: (A) Arterial input function determination, (B) ROI sketching, and (C) Parameter calculation and pseudo-color map generation. DCE: dynamic contrast-enhanced (MRI); ROI: region of interest.


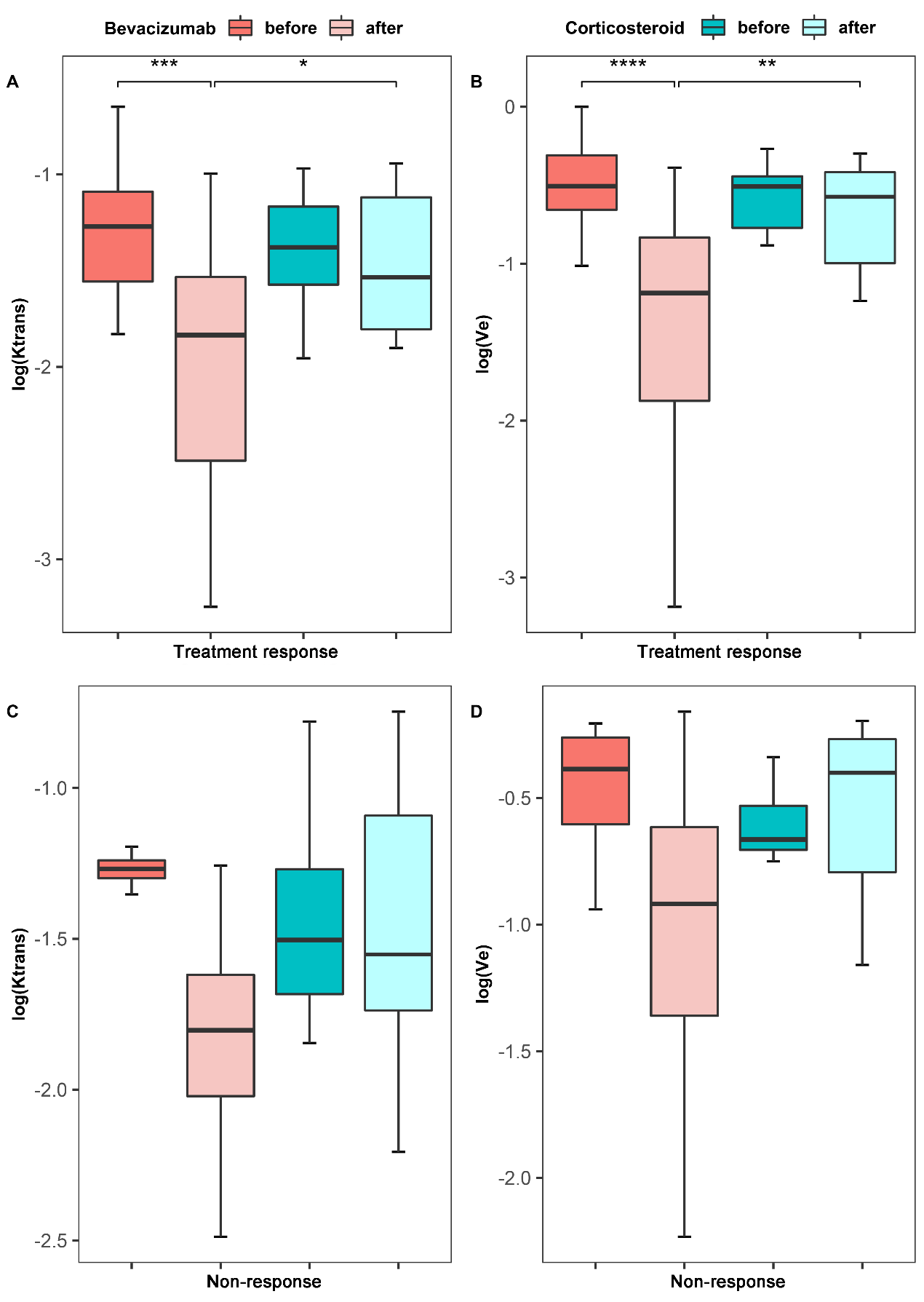


**SUPPLEMENT FIGURE 2** (A-B) Alteration of DCE-derived K^trans^ (A) and v_e_ (B) levels of treatment response cases in two treatment. (C-D) Change of DCE-derived K^trans^ (C) and v_e_ (D) levels of non-response cases in two treatment.
